# YOLO-ACBG: an enhanced deep learning model for precision monitoring of wheat stripe rust using different vegetation indices

**DOI:** 10.3389/fpls.2026.1849651

**Published:** 2026-06-30

**Authors:** Fusheng Yu, Xuanyuan Tang, Qi Liu, Muzaipaer Maimaiti, Jing Chen

**Affiliations:** 1 Key Laboratory of the Pest Monitoring and Safety Control of Crops and Forests of the Xinjiang Uygur Autonomous Region, College of Agronomy, Xinjiang Agricultural University, Urumqi, China; 2 Key Laboratory of Prevention and Control of Invasive Alien Species in Agriculture & Forestry of the North-Western Desert Oasis, Ministry of Agriculture and Rural Affairs, Urumqi, China

**Keywords:** BiLevelRoutingAttention, NDVI, vegetation index, wheat stripe rust, YOLO-ACBG model

## Abstract

Wheat stripe rust (WSR) is a fungal disease that significantly impacts wheat yield and quality. Pathogen-induced pigment degradation and cellular structural damage cause specific reflectance variations, notably, an increase in the red band due to reduced chlorophyll absorption and a concurrent decrease in the near-infrared (NIR) band caused by mesophyll tissue collapse. These physiological changes drive nonlinear shifts in spectral vegetation indices, which can serve as effective indicators for precise disease monitoring. These physiological changes drove nonlinear shifts in spectral vegetation indices, which can serve as effective indicators for precise disease monitoring. To enable precise monitoring of wheat stripe rust in the field, this study constructed 13 datasets corresponding to specific vegetation indices and performed a comparative analysis, identifying Normalized Difference Vegetation Index (NDVI) as the optimal index for detecting wheat stripe rust. The results on different vegetation index datasets demonstrate that the mean Average Precision at IoU threshold 0.5 (mAP@50) of NDVI achieved 92.11%, which is 6.06% higher than RGB. To further improve model performance in identification by fully leveraging information from vegetation indices, this study proposed a model named YOLO-ADown+ConvFormer+BiLevelRoutingAttention+CGLU (YOLO-ACBG), improved from the YOLO-ADown+ConvFormer (YOLO-AC) model, which demonstrates better performance in WSR monitoring and can be deployed on edge device. This model introduced a Bi-Level Routing Attention mechanism and further integrates a convolutional gated linear unit into the C3K2_ConvFormer module. The results on NDVI datasets demonstrate that YOLO-ACBG achieves an mAP@50 of 94.12%, which is 2.01% higher than YOLO-AC; the recall is 88.59%, representing a 3.83% improvement over YOLO-AC. These results suggest that the YOLO-ACBG model can precisely monitor wheat stripe rust in the field, providing guidance for the precise prevention and control of plant disease.

## Introduction

The stable production of wheat is essential for safeguarding global food security (Liu et al., [[NoYear]]c). Wheat stripe rust, caused by *Puccinia striiformis* f. sp. *tritici* (*Pst*), is a significant disease that has long threatened the safe production of wheat in China (Liu et al., [[NoYear]]b). Additionally, it was a cross-regional airborne disease with the characteristics of wide distribution, strong prevalence, and high incidence (Zeng and Yang, 1986). Wheat stripe rust, which triggered eight large-scale epidemics from 1950 to 2020 and caused a cumulative wheat production loss of over 13.89 million tons, is one of the main factors constraining the stable increase of wheat production in China (Gao et al., 2021). Traditional crop disease investigation primarily depended on field sampling conducted by personnel, which was time-consuming, labor-intensive, and imposed a substantial workload. It was difficult to meet the demands of large-scale monitoring and forecasting.

Due to the mobility and adaptability, Unmanned Aerial Vehicle (UAV) platforms were employed to acquire wheat canopy spectra (Wang et al., 2025). Vegetation indices (VIs) have evolved into a primary tool for monitoring crop stress, with extensive applications ranging from fundamental academic research to large-scale field diagnostics (Huang et al., 2026). Moreover, vegetation indices play a central role in estimating crop physiological and biochemical parameters, particularly through the integration of various remote sensing platforms with different modeling approaches (Tian et al., 2026). Fundamentally, this detection relies on a robust link between plant pathology and canopy spectral response: disease-induced chlorophyll loss directly increases reflectance in the red band, while the disruption of cellular structures causes a significant decrease in the near-infrared (NIR) region. In wheat stripe rust detection, combining pigments and spectral indices from UAV hyperspectral imagery achieved 78.1% and 81.3% accuracy at 7 and 16 DPI, outperforming single-method approaches and enhancing early detection (Guo A. et al., [[NoYear]]). In quantifying wheat stripe rust severity, a novel spectral index (YROI) integrating spore response and PROSPECT-D achieved superior accuracy (*R*^2^ = 0.822), enabling quantitative disease assessment beyond traditional qualitative methods (Ren et al., 2021). Wheat stripe rust monitoring at the incubation stage, optimizing spectral features using pseudo-absorption coefficient derivatives, achieved 94.33% accuracy (925–1,075 nm), demonstrating that appropriate spectral transformation was critical for early disease detection (Liu et al., [[NoYear]]a). Collectively, these studies demonstrated that vegetation indices not only play a vital role in monitoring crop physiological and biochemical parameters but also serve as a core source of features for crop disease identification.

As an advanced subset within machine learning, deep learning (DL) (Maimaitijiang et al., 2026) employs multilayered network topologies to automatically delineate and categorize the regions of interest (ROIs). By extracting hierarchical representations directly from massive data volumes, DL transcends the limitations of classical algorithms, delivering superior precision, faster execution, and exceptional scalability. Zhang et al (Zhang T. et al., [[NoYear]]). developed an Ir-UNet algorithm using UAV multispectral images to address irregular boundaries for accurate wheat stripe rust detection, achieving over 97% overall accuracy. Deng et al. (2024) proposed RustQNet, which fuses Red Green Blue (RGB)/multispectral/VI data with mutual information minimization for precise early wheat stripe rust detection. Shant et al (Kumar et al., 2025). compared traditional ML and Convolutional Neural Network (CNN) models for early wheat stripe rust detection, proposing an MCNN integrated with SVM principles that achieved 98% accuracy on two datasets, outperforming standard CNN by 1.2% with higher efficiency. The experimental results presented above confirm that deep learning techniques not only adapt to multiple data types but also excel in wheat stripe rust identification. This capability enables effective disease detection and offers a rapid and precise tool for monitoring wheat stripe rust in the field.

In recent years, diverse attention mechanisms have been widely applied to boost the effectiveness of deep learning models. In line with the working mechanism of human visual attention, these techniques empower deep networks to conduct selective perception and weighted processing on the input information (Guo M-H. et al., [[NoYear]]). In the field of computer vision, attention mechanisms can greatly improve the efficiency and accuracy of perceptual information processing. To address the issue of information overload, attention mechanisms are used as a framework for efficient resource allocation. Nigam et al. (2024) proposed EfficientNet B0-CBAM, which incorporates the Convolutional Block Attention Module (CBAM) attention module and achieved 98.70% accuracy on WheatRust21 by effectively highlighting infected leaf regions. Wen et al. (2024) proposed an MnasNet-SimAM model, enhanced with the SimAM attention mechanism, which effectively detected multiple wheat diseases in complex field environments, providing a practical solution for rapid and reliable agricultural disease management. Wang et al (Wang and Yu, 2025). proposed YOLOv8s-SCR with SE attention, enhancing focus on critical lesion features while maintaining a lightweight design and reducing parameters by 21.02%, while boosting detection accuracy from 84.6% to 90.5% for wheat scab. ; Ma et al. (2024) enhanced YOLOv8 by integrating a Global Attention Mechanism (GAM) to improve semantic and spatial feature fusion, which boosted detection precision, reduced the model size by 11.3%, and increased precision and mAP by 4.5% and 1.9%, respectively, thereby enabling efficient deployment on mobile devices for wheat disease detection. These prior investigations collectively underscore the efficacy of attention modules in improving the performance of automated crop disease detection frameworks. Driven by the specific morphology of wheat stripe rust, we integrated a Convolutional Gated Linear Unit (CGLU) into the C3K2_ConvFormer module to improve complex disease detection. In UAV images, rust lesions typically appear as faint, disjointed streaks obscured by severe canopy clutter. While the baseline C3K2_ConvFormer efficiently models global context, the added CGLU operates as an adaptive spatial filter. It selectively magnifies subtle pathological textures and attenuates environmental interference, ensuring this architectural design is explicitly informed by the physical traits of the pathogen.

In this study, a total of 13 datasets, each derived from different vegetation indices, were constructed and systematically evaluated to determine the most effective index for wheat stripe rust identification. Moreover, the YOLO-AC model was proposed to improve the YOLO-ACBG segmentation efficiency for wheat stripe rust by introducing the BRA attention mechanism and integrating CGLU into C3K2_ConvFormer to construct the C3K2_ConvFormerCGLU module. The results enhance wheat stripe rust management by improving segmentation efficiency with the YOLO-ACBG model and fully exploiting vegetation index information in images to further boost segmentation outcomes, providing a powerful tool for smart agriculture and ensuring the security of wheat production.

## Materials and methods

### Experimental site

**Figure 1:**
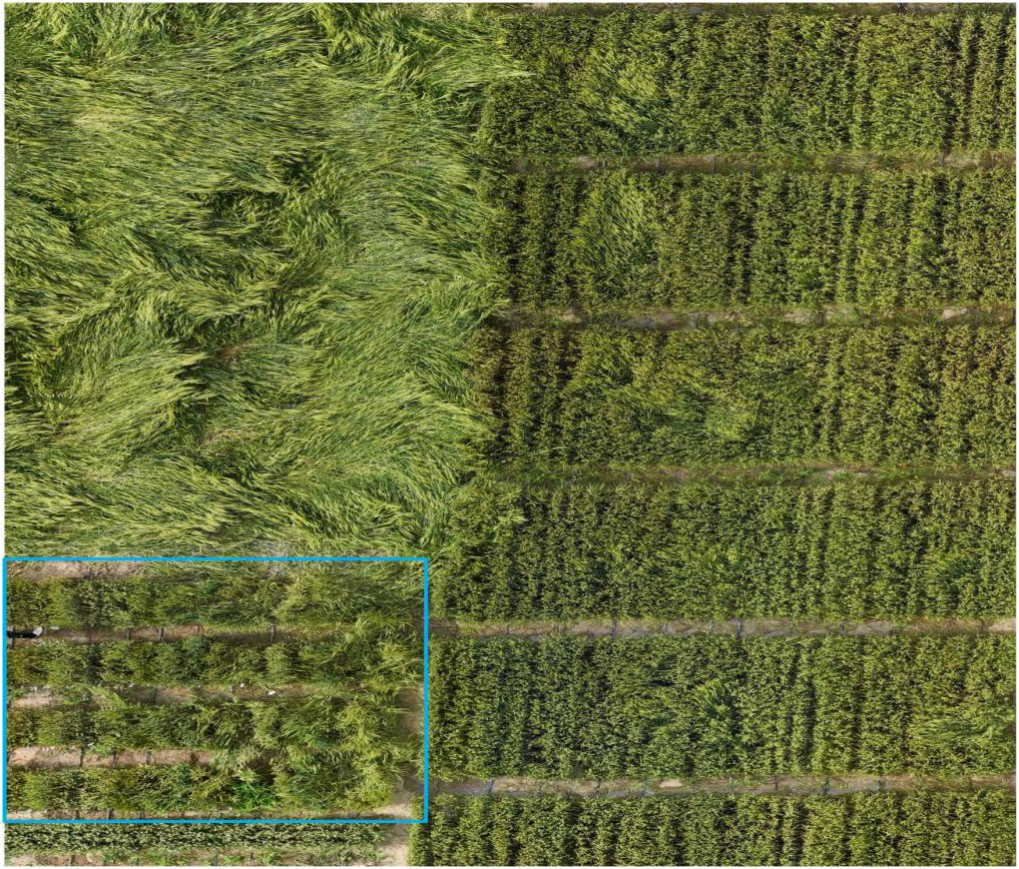
Experimental site.

A 2023–2024 field trial was implemented in Qapqal Xibe Autonomous County of Xinjiang (43°39′50.256″ N, 80°55′0.516″ E). As depicted in **Figure 1**, the blue-colored area indicates the inoculation zone for field tests, and the total experimental area reached 1,000 m^2^. The experimental materials planted in the field included Mingxian 169 (MX169), a genotype highly susceptible to wheat stripe rust, and the Xindong series winter wheat, a major commercial wheat variety in Xinjiang. The inoculum was prepared by suspending *Pst* spores in a solution with 0.02% Tween-80, and the final spore concentration was controlled at 900 mg/L. The inoculation method of wheat stripe rust in the field was described by Zhang et al (Zhang M. et al., [[NoYear]]).

### Experimental methods

#### Multispectral data collection

The image acquisition was performed by a DJI Phantom 4 Multispectral drone. The drone has five sensors, which respectively capture the blue, green, red, red-edge, and near-infrared bands (**Table 1**).

**Table 1:** DJI phantom 4 multispectral bands.

Bands	Center wavelength (nm)	Bandwidth (nm)
Blue	450	434–466
Green	560	544–576
Red	650	634—666
Red edge	730	714—746
NIR	840	814—866

#### Calculation of VIs

The progression of fungal infection induces severe structural degradation and pigment depletion within wheat leaves. Given the widespread application of vegetation indices in estimating crop vitality and biomass, we identified and adopted a series of disease-sensitive indices from existing literature to facilitate the continuous monitoring of this pathological process. Therefore, these selected vegetation indices were expected to provide a scientific basis for discerning wheat stripe rust, as shown in **Table 2**.

**Table 2:** Selection of vegetation indices.

VIs	Formula
NDVI (Rouse, 1974)	(NIR − RED)/(NIR + RED)
GNDVI (Daughtry et al., 1992)	(NIR − GREEN)/(NIR + GREEN)
OSAVI (Rondeaux et al., 1996)	(NIR − RED)/(NIR + RED + 0.16)
NDRE (Fitzgerald et al., 2010)	(NIR − RED EDGE)/(NIR + RED EDGE)
GRVI (Gitelson et al., 2003)	(GREEN − R)/(GREEN + R)
RDVI (Roujean and Breon, 1995)	(NIR − R)/
CIre (Gitelson et al., 2005)	(NIR/RED EDGE) − 1
GI (Kauth and Thomas, 1976)	NIR/GREEN
RVI (Pearson and Miller, 1972)	NIR/R
DVI (Richardson and Wiegand, 1977)	NIR − R
GRRI (Motohka et al., 2010)	GREEN/R
GCVI (Broge and Leblanc, 2001)	(NIR/GREEN) − 1

#### Data preprocessing and labeling

UAV imagery capturing the healthy, latent, and symptom appearance stages of wheat stripe rust was collected at 5 and 12 m altitudes from April to June in 2024 under sunny and windless conditions with plenty of sunlight. The study utilized Pix4Dmapper (v4.3.33) for orthomosaic generation and reflectance calibration, followed by ArcGIS Pro 2.5 to calculate VIs, remove shadows, and composite RGB + VIs images. To prevent spatial autocorrelation, 400 nonoverlapping regional images were extracted and strictly partitioned into geographically independent training (70%), validation (20%), and test (10%) sets.

Guided by synchronous field survey data, a plant protection specialist used LabelMe to manually delineate polygonal boundaries for “healthy” and “rust” instances, excluding background elements. The annotations were exported into the YOLO segmentation format, and all images were resized to a consistent spatial dimension. Finally, standard geometric flips were applied to yield 1,600 images per dataset, and dynamic Mosaic augmentation was integrated during model training to further enhance robust feature learning.

#### YOLOV11-seg algorithm

**Figure 2:**
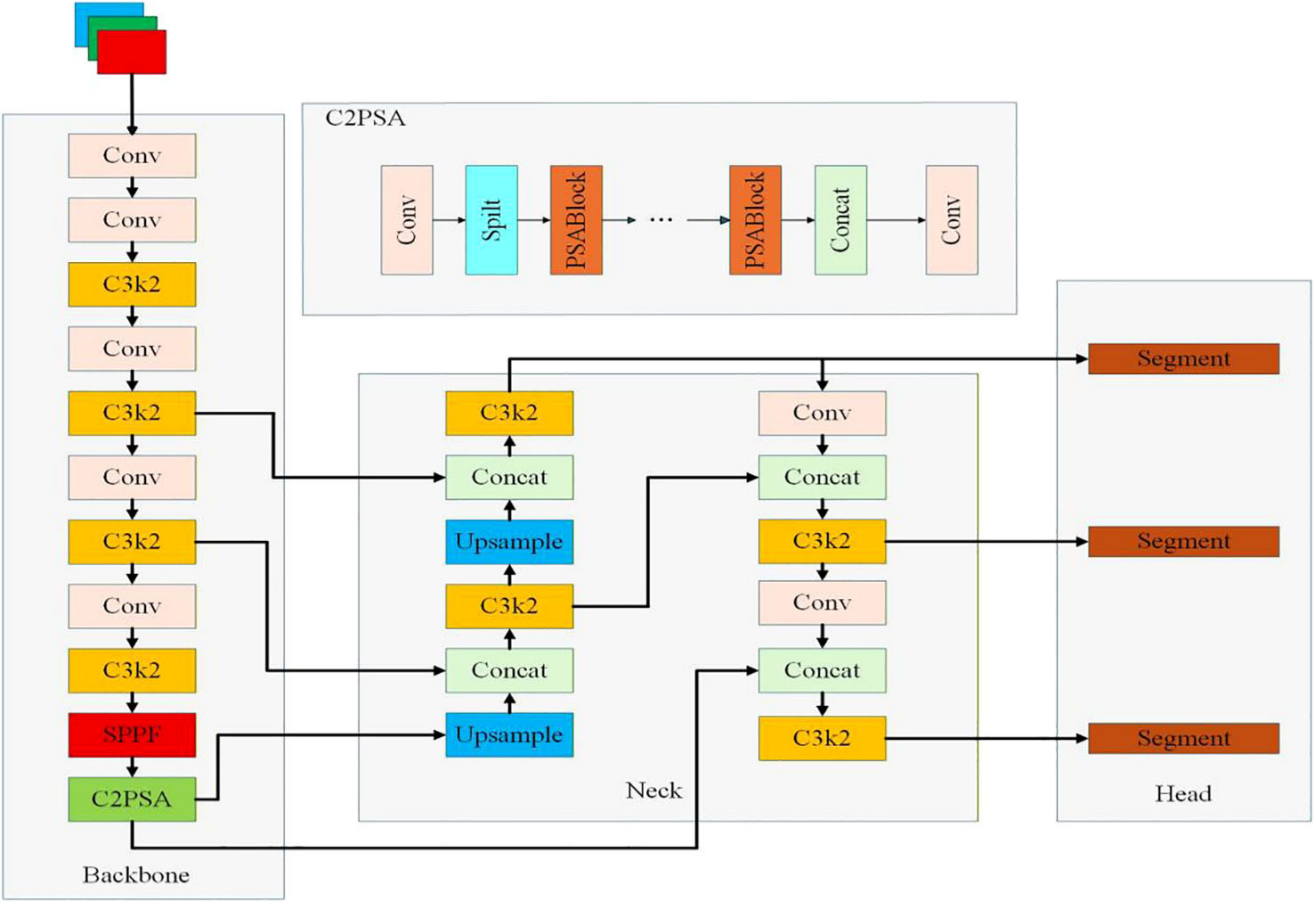
The network structure of the YOLOV11-seg algorithm.

Functioning as an end-to-end, single-pass architecture, the You Only Look Once (YOLO) paradigm bypasses complex region proposals by directly regressing spatial coordinates and class probabilities, thereby drastically accelerating visual recognition. YOLOv11 (**Figure 2**) adopts a lightweight design with depthwise separable convolutions for edge deployment. In most versions, model suffixes—n (nano), s (small), m (medium), l (large), and x (kLarge)—indicate increasing size and accuracy, allowing a trade-off between speed and precision for different deployment needs.

### Model construction

#### Convolutional Gated Linear Unit

**Figure 3:**
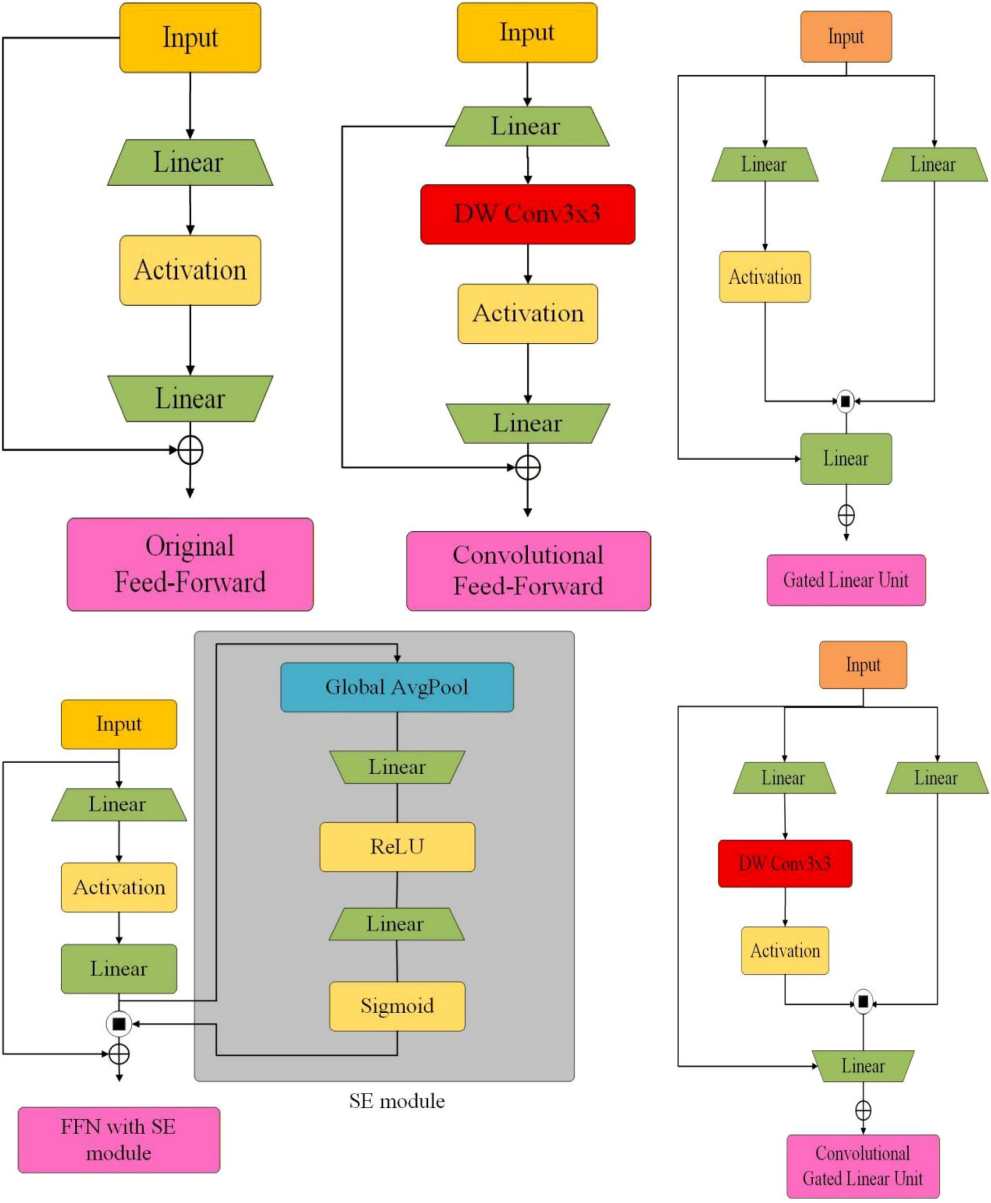
CGLU structure diagram.

Detection of wheat stripe rust in field conditions is challenging due to the subtle spectral and textural variations between healthy leaves and early-stage lesions. To address this, we integrated CGLU (Shi, 2023). This modification (**Figure 3**) transitions the unit from a simple gate to a feature-aware attention mechanism, enabling the network to selectively amplify subtle rust textures while filtering out environmental noise from the field background.

#### Bi-Level Routing Attention mechanism

**Figure 4:**
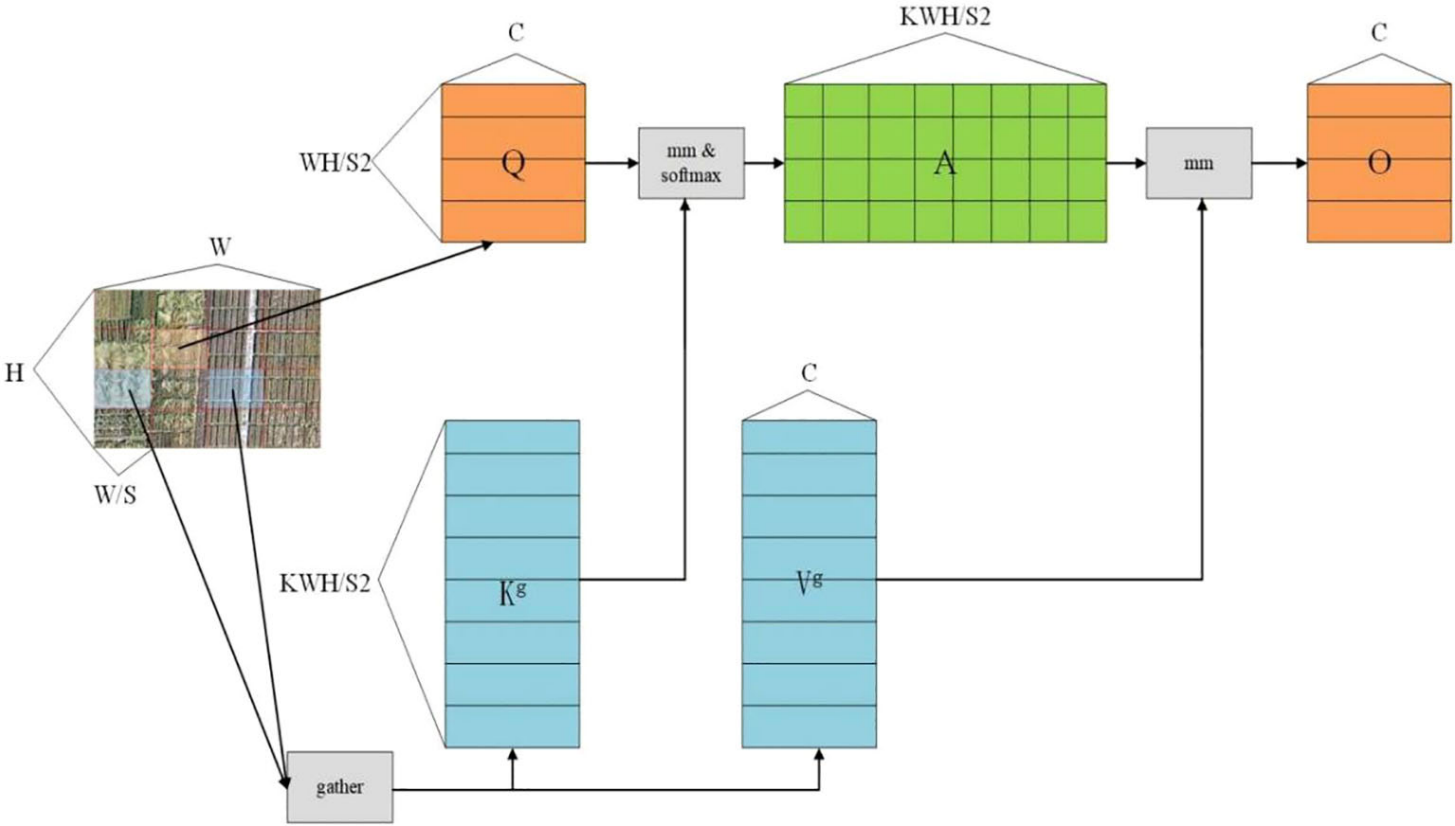
BRA attention mechanism structure.

Bi-Level Routing Attention (Zhu et al., 2023) (BRA) is an attention mechanism designed for deep learning models that effectively captures critical information when processing complex tasks. The core idea of this mechanism is to analyze and process input data in a hierarchical manner, thereby improving model performance and efficiency. BRA (**Figure 4**) introduces a hierarchical structure that enables multilevel analysis of input data. This hierarchical attention computation reduces unnecessary computational steps while improving adaptability to diverse input conditions, allowing the model to more effectively capture critical features in images and thereby improving classification and detection accuracy (Guo Z. et al., [[NoYear]]).

The mechanism aggregates key–value pairs and employs sparse operations to bypass calculations in the less relevant regions, resulting in savings in terms of parameters and computational resources.

### Evaluation indicators

To comprehensively assess the model’s performance, several evaluation metrics were employed, including precision, recall, mAP@50, Giga Floating-Point Operation (GLOPs), and parameter count. Precision measures the accuracy of positive predictions by calculating the ratio of correctly identified masks to the total number of predicted masks, with its mathematical expression provided in **Equation 1**. Recall, on the other hand, indicates the model’s ability to detect all relevant instances by comparing the number of correct predictions to the total ground truth masks, as defined in **Equation 2**. Average precision (AP) serves as a definitive benchmark for assessing detection efficacy. By mapping precision against recall to form a continuous trajectory, AP captures the overall model performance across varying confidence cutoffs, with its calculation specified in **Equation 3**. Finally, the primary detection metric was mask mAP@50, denoted as mAP@50 (M). A predicted mask was considered correct when its mask IoU with the corresponding ground-truth mask exceeded 0.50. It is important to note that, since the task is instance segmentation, the reported mAP@50 metric specifically evaluates the precision of the pixel-level segmentation masks rather than bounding boxes, reflecting the model’s ability to delineate accurate lesion boundaries. The computation of mean Average Precision across all categories was presented in **Equation 4**:

(1) $$\mathrm{Pr}ecision=TP/\left(TP+FP\right)$$

(2) Recall=TP/TP+FN

(3) $$AP=\int_{0}^{1}P\left(R\right)dR$$

(4) $$mAP@50=\frac{1}{n}\sum_{i=1}^{n}APi$$

Where TP is the number of positive samples correctly predicted, FP is the number of negative samples incorrectly predicted, TN is the number of negative samples correctly predicted, and FN is the number of positive samples incorrectly predicted.

### Experimental environment

The entire experiment was executed on a Linux operating system using the PyTorch 2.0.0 (GPU edition) framework. The software environment consisted of CUDA 11.8 and Python 3.8. The computational resources included a 22 vCPU AMD EPYC 7T83 64-Core Processor for dataset training and an RTX 4090 GPU (24 GB VRAM) for accelerated computing. The training hyperparameters were set as follows: number of epochs = 100, batch size = 16, initial learning rate (lr0) = 0.01, optimizer = SGD, input image size = 640 × 640 pixels, and random seed = 0.

## Results

### The performance of different vegetation indices

The results of 13 different datasets on wheat stripe rust (WSR) recognition are shown in **Table 3**. NDVI achieved the highest mAP@50 (92.12%) and the highest precision (94.23%) among the VI datasets. Therefore, the NDVI datasets can be employed for precise WSR identification, providing high-quality data for subsequent model improvement.

**Table 3:** Performance results of different vegetation indices.

VIs	Precision (%)	Recall (%)	mAP@50 (%)
RGB	89.56	79.16	86.06
CIre	90.42	79.93	86.72
DVI	92.14	84.57	90.18
GCVI	87.93	80.45	87.25
GI	92.82	82.57	90.05
GNDVI	88.66	76.61	87.61
GRRI	93.03	79.82	87.62
GRVI	91.88	81.18	87.89
NDRE	89.23	74.62	83.82
NDVI	94.23	84.76	92.12
OSAVI	91.08	78.82	85.67
RDVI	91.25	79.67	87.61
RVI	90.69	79.26	87.14

**Figure 5:**
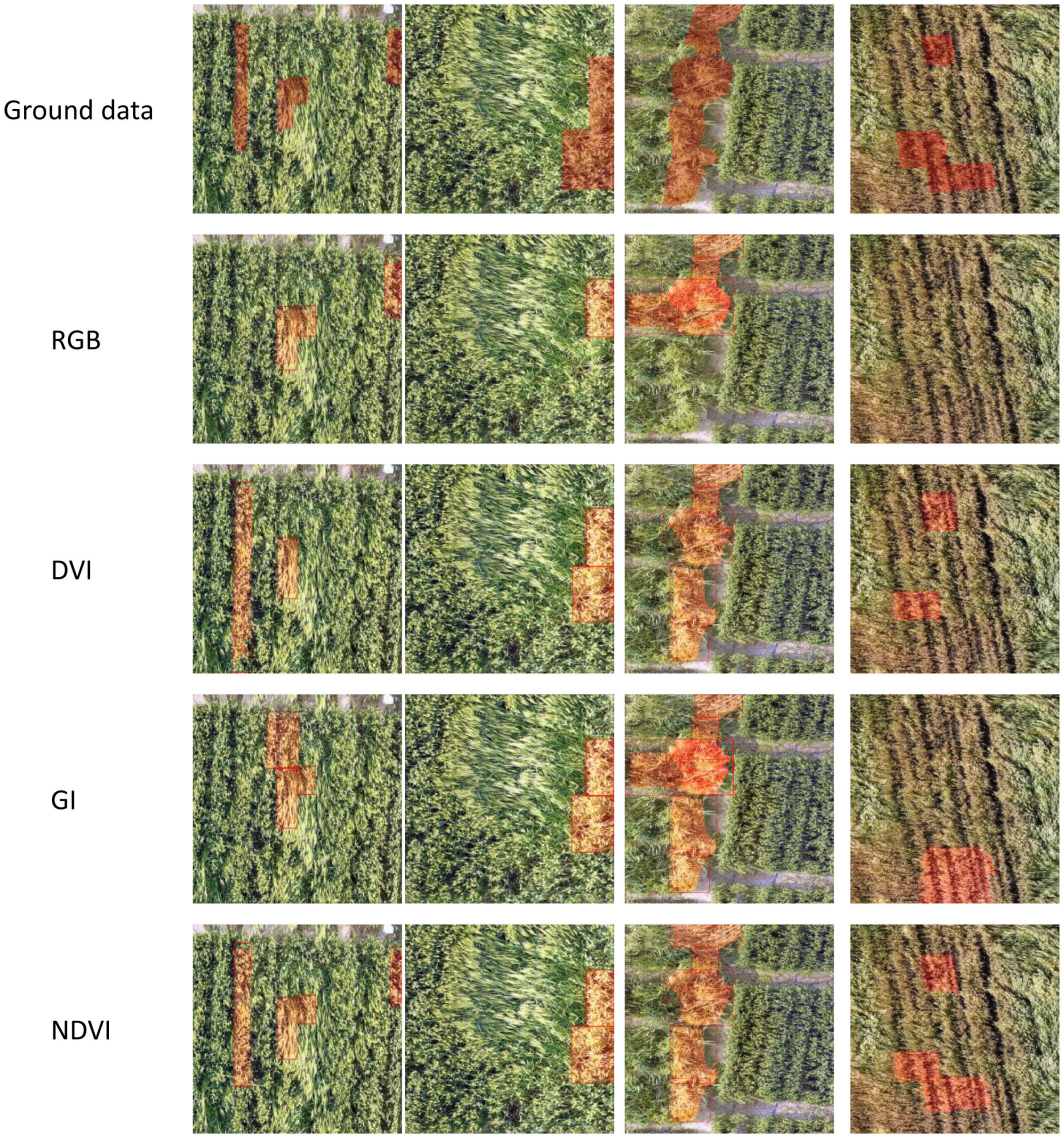
Comparison of test results for different vegetation indices.

According to **Table 3**, models with mAP@50 above 90% were selected for further analysis on the test sets. The results are shown in **Figure 5**. Comparative analysis across different test plots revealed that RGB images alone resulted in missed detection and suboptimal segmentation performance in regions with indistinct features. Among all tested inputs, images augmented with NDVI yielded the best segmentation results, enabling effective identification of various disease-affected areas and achieving precise segmentation, which illustrates that the incorporation of vegetation index information effectively reduces missed detections and improves segmentation performance. Consequently, the NDVI datasets can be employed for segmentation tasks, offering directions for subsequent model improvements.

### Comparison results of models with different attention mechanisms

In **Table 4**, the model with the BRA mechanism demonstrated improved segmentation performance. Compared with the YOLO-AC model, the BRA model showed improvements of 0.13% in precision, 0.72% in recall, and 0.17% in mAP@50, while maintaining the original level of computational cost and striking an optimal balance between performance and efficiency.

**Table 4:** Comparison results of different attention mechanisms.

Models	Precision (%)	Recall (%)	mAP@50 (%)	GFLOPs	Parameters/106 (M)
YOLO-AC	94.23	84.76	92.11	123.9	23.18
YOLO-AC + DAttention (Xu et al., 2026)	93.33	86.89	91.48	124.7	24.24
YOLO-AC + C2DA	92.63	87.91	91.25	125.1	24.77
YOLO-AC + SimAM (Cheng et al., [[NoYear]])	93.41	86.78	91.35	123.9	23.18
YOLO-AC + BRA	94.36	85.46	92.28	124.9	24.23

### Ablation experiment

As shown in **Table 5**, the ablation experiment results indicate that the YOLO-AC model integrating the C3k2_ConvFormerCGLU and BRA achieved the best performance in wheat stripe rust detection. The baseline model, YOLO-AC, achieved a precision of 94.23%, a recall of 84.76%, and an mAP@50 of 92.11%, with 123.9 GFLOPs and 23.18 million parameters. Incorporating the CGLU module (YOLO-AC + CGLU) improved all three accuracy metrics while reducing computational cost and parameter count, whereas adding the BRA module (YOLO-AC + BRA) yielded marginal performance gains but slightly increased computational overhead compared to the baseline. The final model, YOLO-ACBG, which integrated both modules, attained the best overall performance with a precision of 94.78%, a recall of 88.59%, an mAP@50 of 94.12%, and competitive computational efficiency (116.7 GFLOPs, 22.07 million parameters). These results confirm that the synergistic combination of the CGLU and BRA modules significantly enhances detection performance while maintaining practical feasibility for remote sensing applications.

**Table 5:** Results of the ablation experiment.

Models	Precision (%)	Recall (%)	mAP@50 (%)	GFLOPs	Parameters/106 (M)
YOLO-AC	94.23	84.76	92.11	123.9	23.18
YOLO-AC + CGLU	94.48	84.99	93.27	105.8	21.02
YOLO-AC + BRA	94.36	85.16	92.28	124.9	24.23
YOLO-ACBG	94.78	88.59	94.12	116.7	22.07

**Figure 6:**
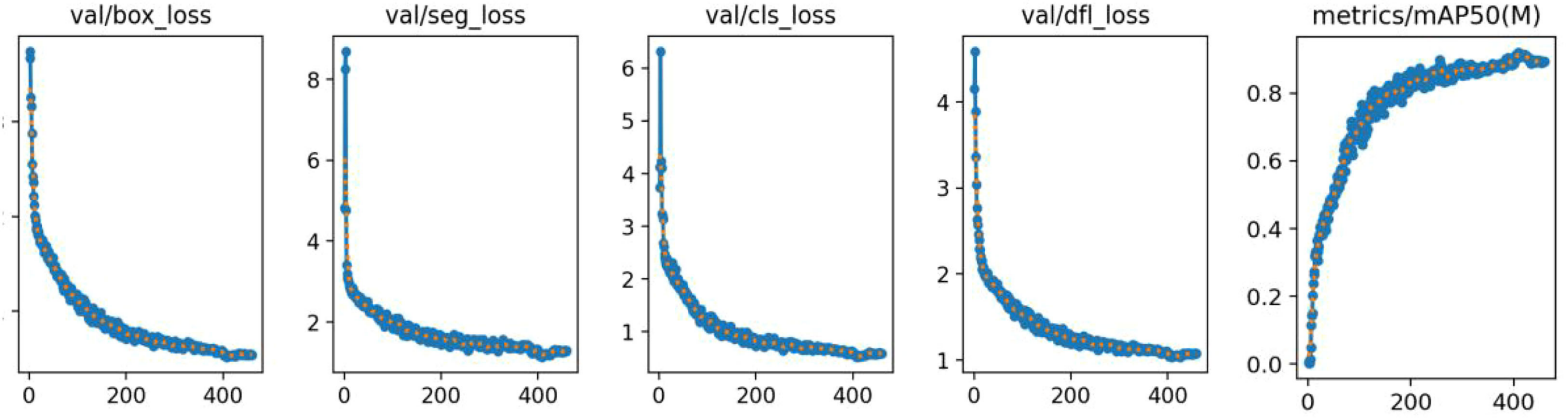
Performance metrics curve of YOLO-ACBG.

**Figure 6** plots the key performance metric curves from the validation set for YOLO-ACBG, which clearly verifies the model’s convergence during training. All validation loss metrics, including val/box_loss, val/seg_loss, val/cls_loss, and val/dfl_loss, exhibited consistent downward trends and eventually stabilized without significant decreases with increasing iterations. Meanwhile, the core evaluation metric metrics/mAP@50(M) showed a decelerating growth rate and ultimately reached its peak value, directly demonstrating that the model’s detection accuracy had achieved an optimal state. These convergent phenomena collectively indicate that the prediction errors of the model on the validation set had dropped to a low level, and the overall detection performance had reached a stable and satisfactory level.

### Comparison results of different segmentation models

As **Table 6** shows, the performance comparison results between YOLO-ACBG and mainstream YOLO-series models on the NDVI dataset validate the superior performance of the proposed model in instance segmentation tasks. YOLO-ACBG outperformed YOLOv11-seg and YOLOv8-seg variants across three core evaluation metrics, achieving a precision of 94.78%, a recall of 88.59%, and an mAP@50 of 94.12%. Meanwhile, this model maintained competitive computational efficiency with only 116.7 GFLOPs of computational complexity and 22.07 million parameters, thereby striking an optimal balance between detection accuracy and computational overhead. The excellent performance of YOLO-ACBG further verified the great potential of spatial-channel dual attention mechanisms in efficient visual perception tasks. These findings collectively provided a novel and effective solution for real-time high-precision instance segmentation applications in agricultural remote sensing scenarios.

**Table 6:** Comparison results with other YOLO algorithms on the NDVI datasets.

Models	Precision (%)	Recall (%)	mAP@50 (%)	GFLOPs	Parameters/106 (M)
YOLO-ACBG	94.78	88.59	94.12	116.7	22.07
YOLOv11l-seg	92.49	81.38	87.41	142.7	27.62
YOLOv11x-seg	90.12	79.86	86.5	319.7	62.05
YOLOv8l-seg	88.57	84.69	88.74	220.8	45.94
YOLOv8x-seg	89.18	81.89	87.59	344.5	71.75

### YOLO-ACBG structure

**Figure 7:**
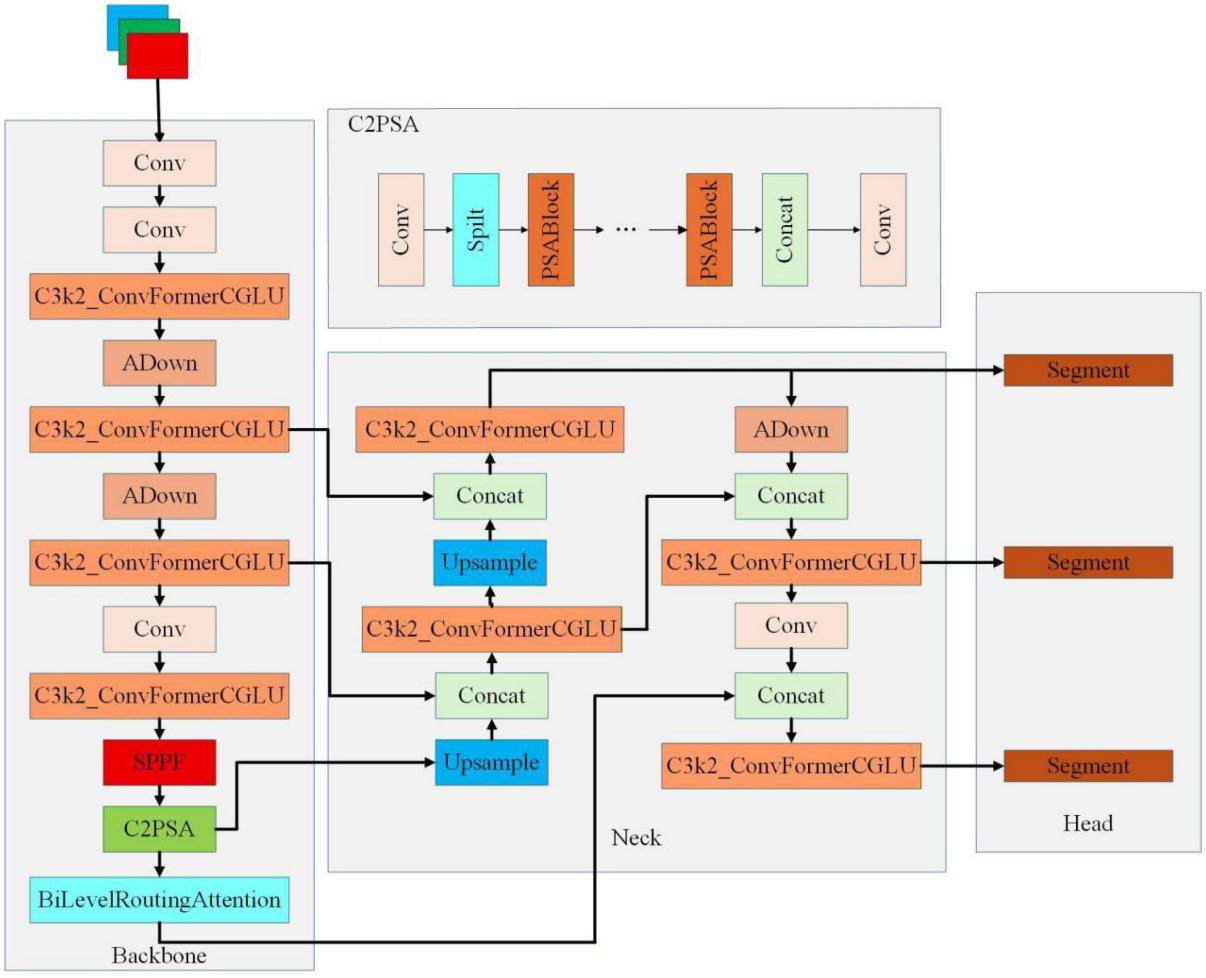
The network structure of the YOLO-ACBG model.

The structure of the YOLO-ACBG model was illustrated in **Figure 7**. In the YOLO-AC backbone network, the C3k2_ConvFormer modules at the second, fourth, sixth, and eighth layers were enhanced by incorporating a gated linear unit, resulting in the C3k2_ConvFormerCGLU module. The BiLevelRoutingAttention mechanism was added after the C2PSA module. In the neck network, the C3k2_ConvFormer modules at the 13th, 16th, 19th, and 22nd layers were replaced by C3k2_ConvFormerCGLU. The head network remained the same.

## Discussion

The experimental results demonstrate that the proposed YOLO-ACBG model achieves superior segmentation performance for WSR by integrating the CGLU module and the BRA mechanism into an NDVI-enhanced input framework. Compared with the baseline YOLO-AC model, YOLO-ACBG yields significant improvements in recall (88.59% vs. 84.76%) and mAP@50 (94.12% vs. 92.11%). To interpret these improvements from a mechanistic perspective, it is necessary to examine the model’s behavior in light of plant pathology and plant physiology.

The observation that NDVI outperforms other spectral indices (**Table 3**; **Figure 5**) is consistent with the pathogenesis of *Pst*. During infection, the pathogen colonizes leaf mesophyll cells, leading to chloroplast degradation, chlorophyll loss, and reduced photosynthetic activity (Guo A. et al., [[NoYear]]; Ren et al., 2021). These physiological changes induce a marked increase in red reflectance due to decreased chlorophyll absorption and a concurrent decrease in near-infrared reflectance owing to disrupted leaf cellular structure and reduced mesophyll scattering. NDVI = (NIR − R)/(NIR + R) directly captures this differential spectral response at the canopy level, rendering it highly sensitive to early and intermediate stages of rust development. By contrast, indices that rely more heavily on red-edge or green bands (e.g., NDRE, GCVI) are less responsive to the specific structural damage caused by *Pst* at the spatial scale of UAV imagery. Therefore, the superior performance of NDVI may be explained by its sensitivity to disease-induced chlorophyll degradation and canopy structural damage, which are known physiological responses to *Puccinia striiformis* infection. Although direct measurements of pigment content or tissue damage were not obtained in this study, this physiological link provides a plausible interpretation for why NDVI outperforms other indices.

The CGLU module replaces conventional feed-forward layers with a convolutional gated linear unit (**Figure 3**) (Shi, 2023). One possible mechanism by which the CGLU module may improve performance is through selective amplification of local spectral-spatial anomalies (e.g., reduced NDVI) that are characteristic of early rust lesions while concurrently suppressing irrelevant background signals such as soil or shadows. However, direct visualization of the gating behavior remains a subject for future investigation. This explains why the YOLO-AC + CGLU variant reduces GFLOPs and parameter count while improving precision and recall (**Table 5**): it achieves physiologically informed feature selection without incurring additional computational cost. Similar advantages have been documented in other agricultural remote sensing tasks, including forest fire detection (Yu et al., [[NoYear]]), juvenile fish phenotyping (Sun et al., 2025), unsound wheat kernel detection (Guo Z. et al., [[NoYear]]), and cotton disease diagnosis. In the context of WSR, CGLU enhances the model’s ability to recognize disease-induced reflectance anomalies at the pixel and superpixel level rather than relying on fine textural details that may not be reliably captured by UAV multispectral sensors.

WSR exhibits a characteristic spatial distribution in the field: infected plants often appear as disease foci or along rows, with severity varying continuously from asymptomatic to severely affected. The BRA mechanism (**Figure 4**) introduces a hierarchical, query-adaptive sparse attention that first coarsens the feature map into regions and then routes only the most relevant region pairs for fine-grained attention (Zhu et al., 2023). From an epidemiological standpoint, this design is particularly advantageous for UAV imagery: the model can first locate potential disease foci (e.g., patches of altered canopy reflectance indicative of infection) and subsequently refine its focus on individual lesion-bearing areas within those patches. A plausible advantage of the BRA mechanism in this context is its ability to focus computational resources on regions with disease-like anomalies across the field, potentially capturing spatial relationships between disease foci without expending capacity on healthy canopy areas. This interpretation is consistent with the observed recall improvement. This accounts for the 0.72% recall improvement over YOLO-AC (**Table 4**): fewer infected areas are missed because the model’s attention is dynamically allocated to regions exhibiting physiological abnormalities (reduced NIR, increased red) at the canopy scale. Compared with other attention mechanisms (DAttention, C2DA, SimAM), BRA achieves a better trade-off because its sparse routing reduces background interference without sacrificing spatial coverage—a critical requirement for detecting patchy disease distribution in large-scale field images.

The final YOLO-ACBG model combines both modules, attaining the best overall performance (precision: 94.78%, recall: 88.59%, mAP@50: 94.12%). This synergy can be interpreted as follows: the CGLU module operates at a local, spectral-spatial level, extracting disease-induced reflectance anomalies (e.g., reduced NDVI values, altered red and NIR channels) within localized image regions. The BRA module operates at a global, spatial-attention level, directing network capacity toward regions where such anomalies are likely to occur based on broader canopy patterns. This two-stage mechanism mimics a practical remote sensing diagnostic strategy: first, identify suspicious patches of canopy discoloration; second, perform refined segmentation within those patches. By embedding this hierarchical perceptual strategy into the deep learning architecture, YOLO-ACBG achieves both high sensitivity (recall) and high precision without relying on ultra-fine textural details that are not reliably resolvable by UAV multispectral sensors.

The practical implications of this study are twofold. First, the use of NDVI-enhanced RGB imagery on a UAV platform enables nondestructive, large-scale WSR monitoring with edge−deployable computational requirements (116.7 GFLOPs, 22.07 million parameters). The fusion of RGB with vegetation indices has been shown to improve farmland classification and disease detection efficiency compared with using either data type alone (Kim et al., 2023). Second, the mechanistic interpretation provided here directly links model improvements to disease physiology, offering a template for designing interpretable deep learning models for other crop diseases (e.g., powdery mildew, *Fusarium* head blight) that involve similar spectral changes. Nevertheless, the current model was trained on data from a single region (Qapqal Xibe Autonomous County) and a single growing season. Future work should evaluate its generalizability across different wheat varieties, growth stages, and environmental conditions. To fundamentally enhance model robustness, systematic adoption of data augmentation (e.g., rotation, cropping, flipping) and model regularization (e.g., Dropout, weight sharing) is recommended (Wen et al., 2025; Yang and Wang, 2025).

In summary, the YOLO-ACBG model improves WSR segmentation from UAV multispectral imagery by aligning its architectural innovations—CGLU for local spectral−spatial feature extraction and BRA for spatially adaptive attention—with the underlying plant physiological and pathological processes of stripe rust infection while respecting the spatial resolution limitations of remote sensing data. This mechanistic alignment explains the consistent performance gains observed in the present experiments.

## Conclusion

In this study, the impact of integrating various vegetation indices with RGB images on wheat stripe rust detection was evaluated. The results demonstrated that the RGB + NDVI image datasets achieved the highest identification performance, with a 92.12% mAP@50, which significantly improved the model’s segmentation capability. The proposed YOLO-ACBG model demonstrated superior performance in wheat stripe rust segmentation, achieving a 94.12% mAP@50, 88.59% recall, and 94.78% precision, representing significant improvements over the baseline YOLO-AC model while effectively utilizing vegetation index information. The integration of the BiLevelRoutingAttention module and the novel C3K2_ConvFormerCGLU structure enabled the model to improve segmentation performance through more effective utilization of vegetation index information while simultaneously enhancing computational efficiency for potential edge deployment. This study proposed an effective segmentation architecture leveraging vegetation indices, which combines improved detection capabilities to support precision agriculture for wheat stripe rust management.

## Data Availability

The raw data supporting the conclusions of this article will be made available by the authors, without undue reservation.
